# Supplementary figures and images for: Migrators within migrators: exploring transposable element dynamics in the monarch butterfly, Danaus plexippus
Source: Mob DNA. 2022 Feb 16;13:5. doi: 10.1186/s13100-022-00263-5 (PMC8848866; doi:10.1186/s13100-022-00263-5)

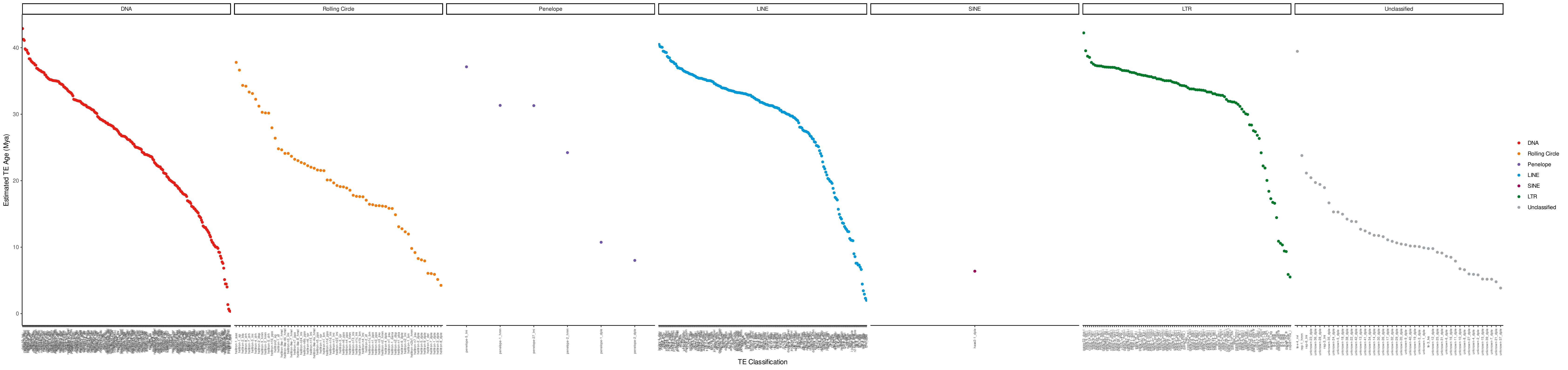

Supplement: Supplementary file 3 — Additional file 3: Fig. S1. Scatter plot illustrating estimated TE age for each TE family. Major TE types are represented by different colours indicated in the key. Elements are ordered from oldest to youngest, within each major type. [file 13100_2022_263_MOESM3_ESM.pdf]

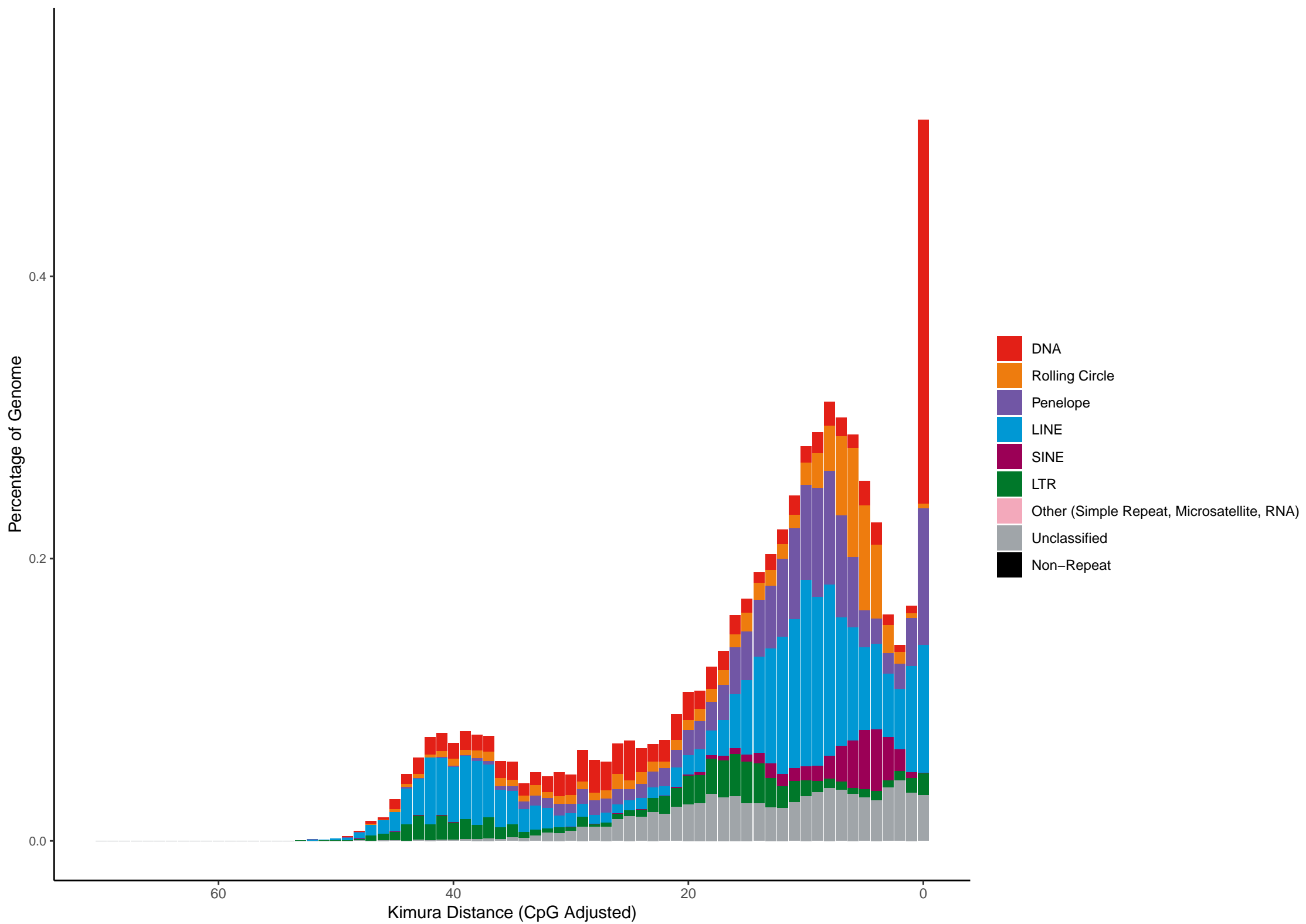

Supplement: Supplementary file 4 — Additional file 4: Fig. S2. Repeat landscapes for the monarch. The x axis indicates the level of Kimura 2-parameter genetic distance observed between TE insertions and their respective consensus sequences in percent. More recent elements are located to the right of the x axis. The y axis indicates the percentage of the genome occupied by TE insertions of each genetic distance class. [file 13100_2022_263_MOESM4_ESM.pdf]

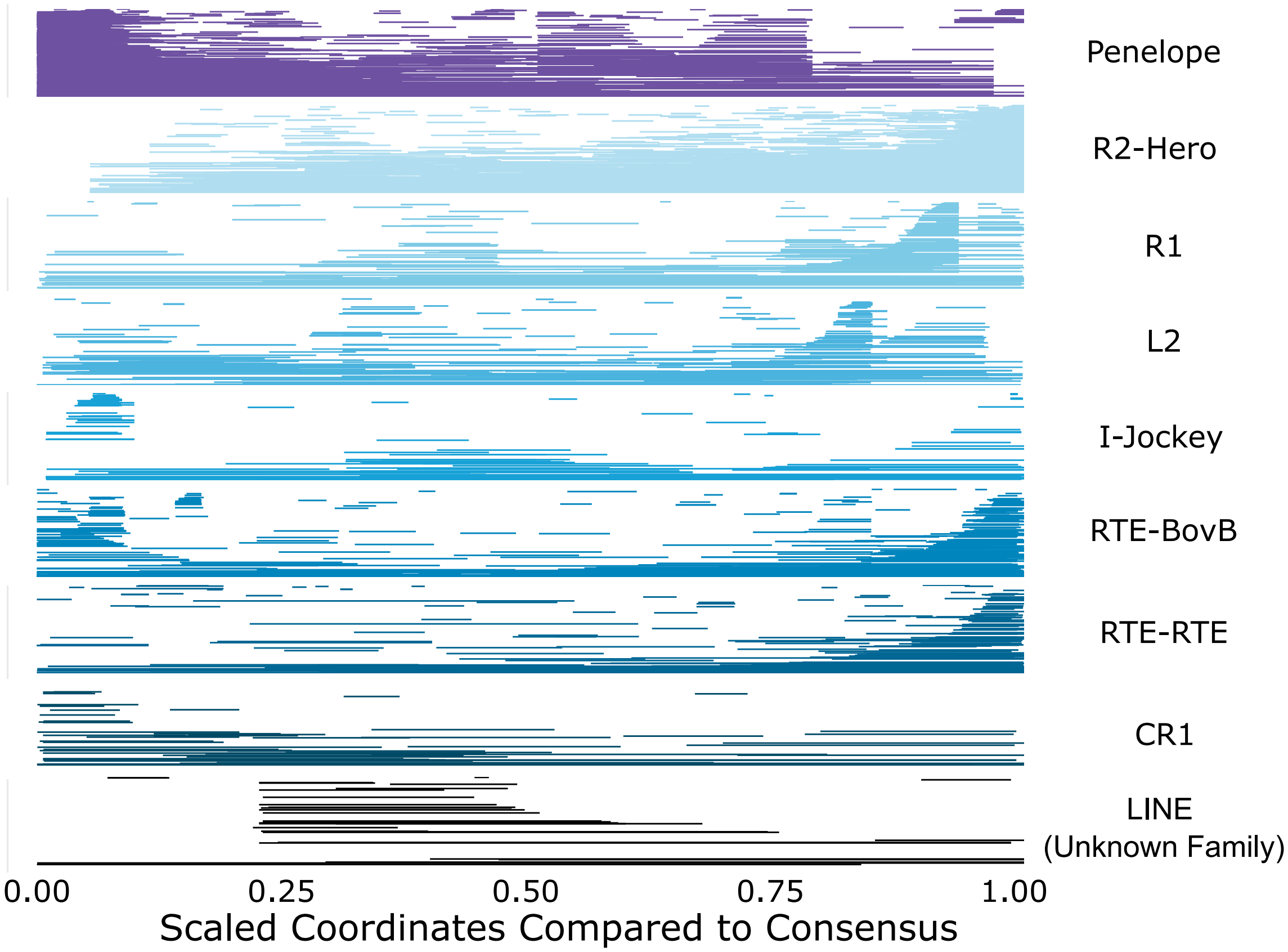

Supplement: Supplementary file 5 — Additional file 5: Fig. S3. Plot illustrating LINE and Penelope fragments annotated in the monarch genome. The x axis indicates normalised start and end coordinates for each element relative to its consensus. Elements are organised from longest to shortest, with short elements found towards the top of the plot. Families are represented by different colours indicated by the key. [file 13100_2022_263_MOESM5_ESM.pdf]

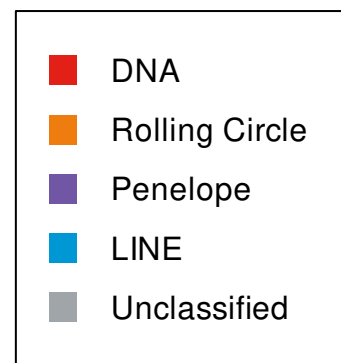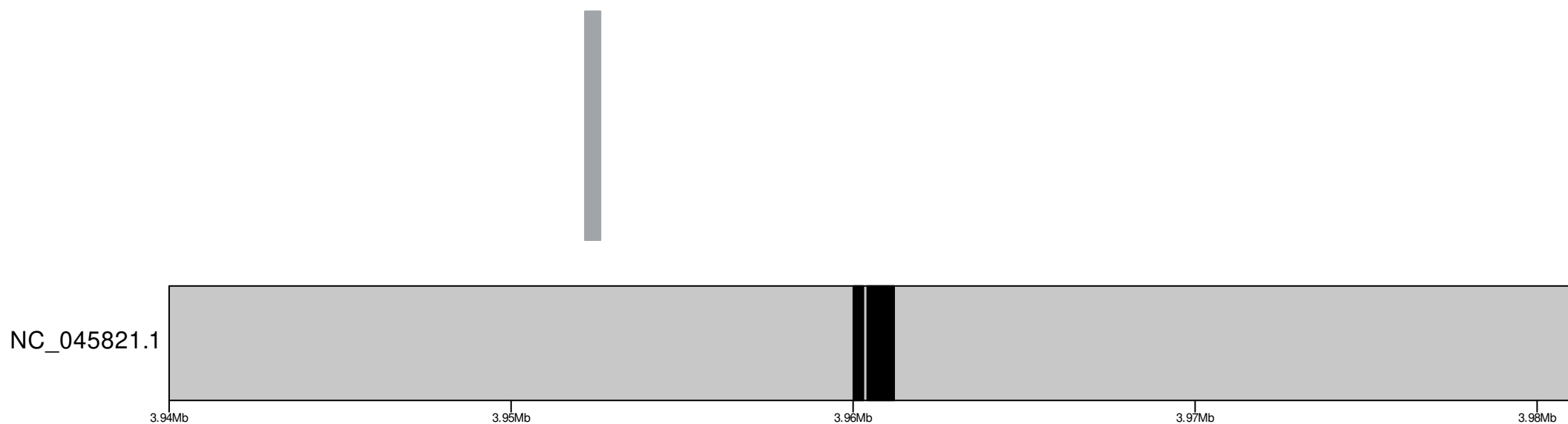

Supplement: Supplementary file 6 — Additional file 6: Fig. S4. Karyoplot of the monarch gene association with colouration, myosin, obtained from MonarchBase under accession DPOGS206617. Main TE types within 20 kb are represented by different colours indicated in the key. [file 13100_2022_263_MOESM6_ESM.pdf]

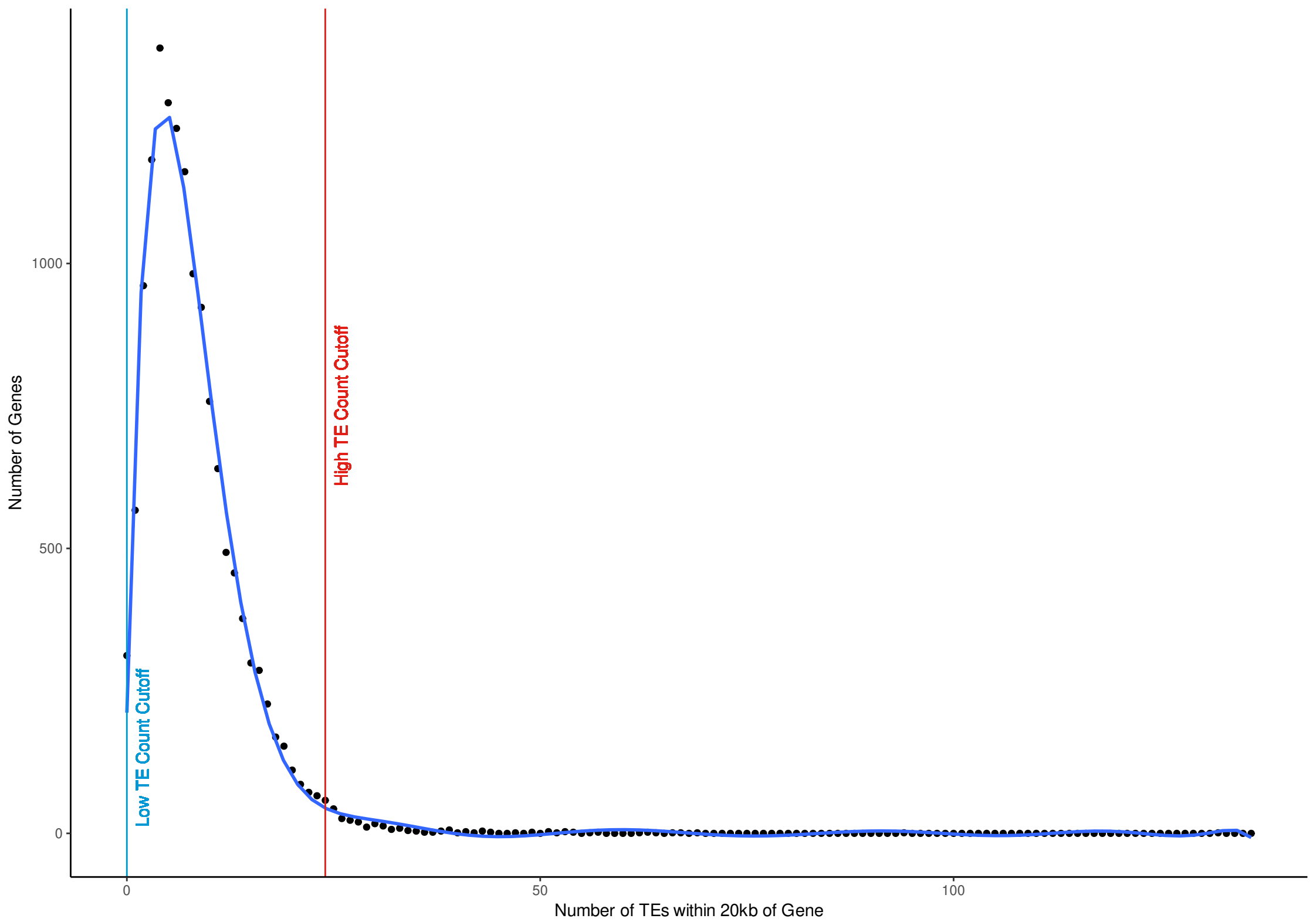

Supplement: Supplementary file 7 — Additional file 7: Fig. S5. Plot illustrating the distribution of TE copy number found with 20 kb of all genes, showing the 2.5 and 97.5% AUC cutoffs used to determine the TE copy number within 20 kb of a given gene for it to be significantly depleted or enriched in comparison to other genes. [file 13100_2022_263_MOESM7_ESM.pdf]

**A**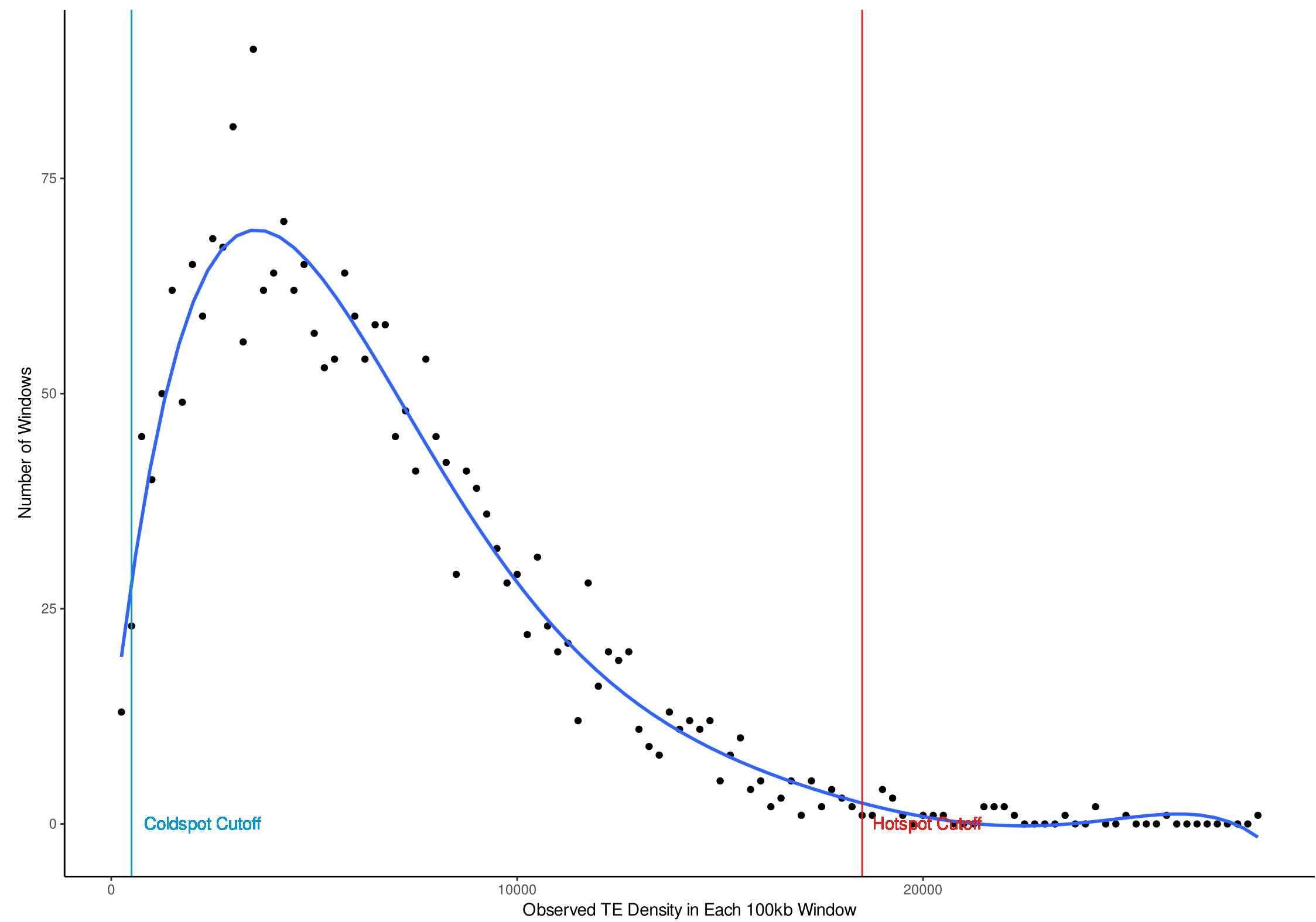**B**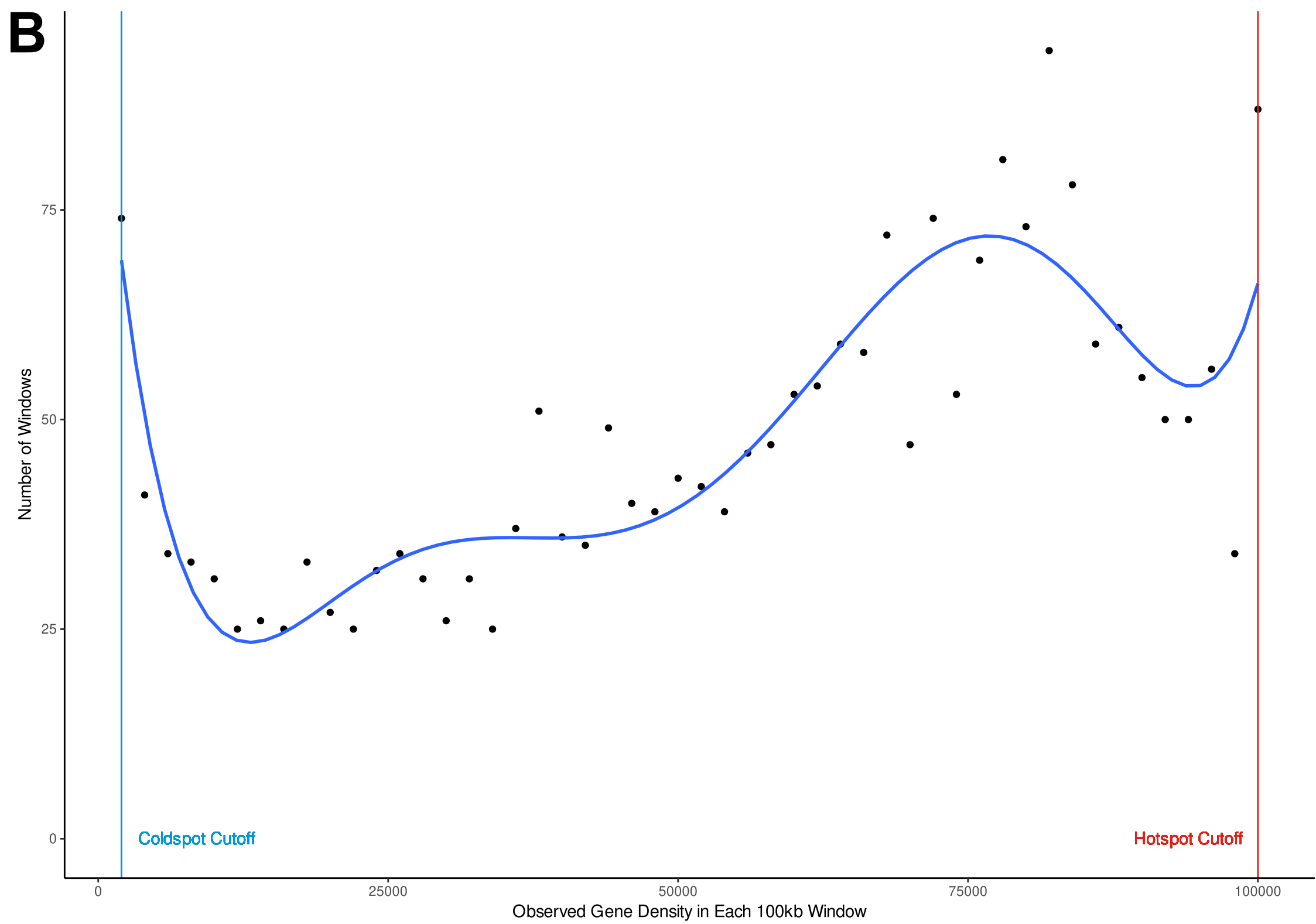

Supplement: Supplementary file 13 — Additional file 13: Fig. S8. Plots illustrating: (A) Distribution of genome windows with TE coverage, with lines showing 1 and 99% AUC cutoffs used to define hotspots and coldspots. (B) Distribution of genome windows with host gene coverage, with lines showing 1 and 99% AUC cutoffs used to define hotspots and coldspots. [file 13100_2022_263_MOESM13_ESM.pdf]
