# Supplementary material for: Migrators within migrators: exploring transposable element dynamics in the monarch butterfly, Danaus plexippus
Source: Mob DNA. 2022 Feb 16;13:5. doi: 10.1186/s13100-022-00263-5 (PMC8848866; doi:10.1186/s13100-022-00263-5)
Supplement: Supplementary file 8 — Additional file 8: Fig. S6. Boxplots illustrating: (A) Normalised TE coverage in 20 kb regions surrounding each immune gene type. (B) Normalised TE count in 20 kb regions surrounding each immune gene type. Main TE types are represented by different colours indicated in the key. [file 13100_2022_263_MOESM8_ESM.pdf]

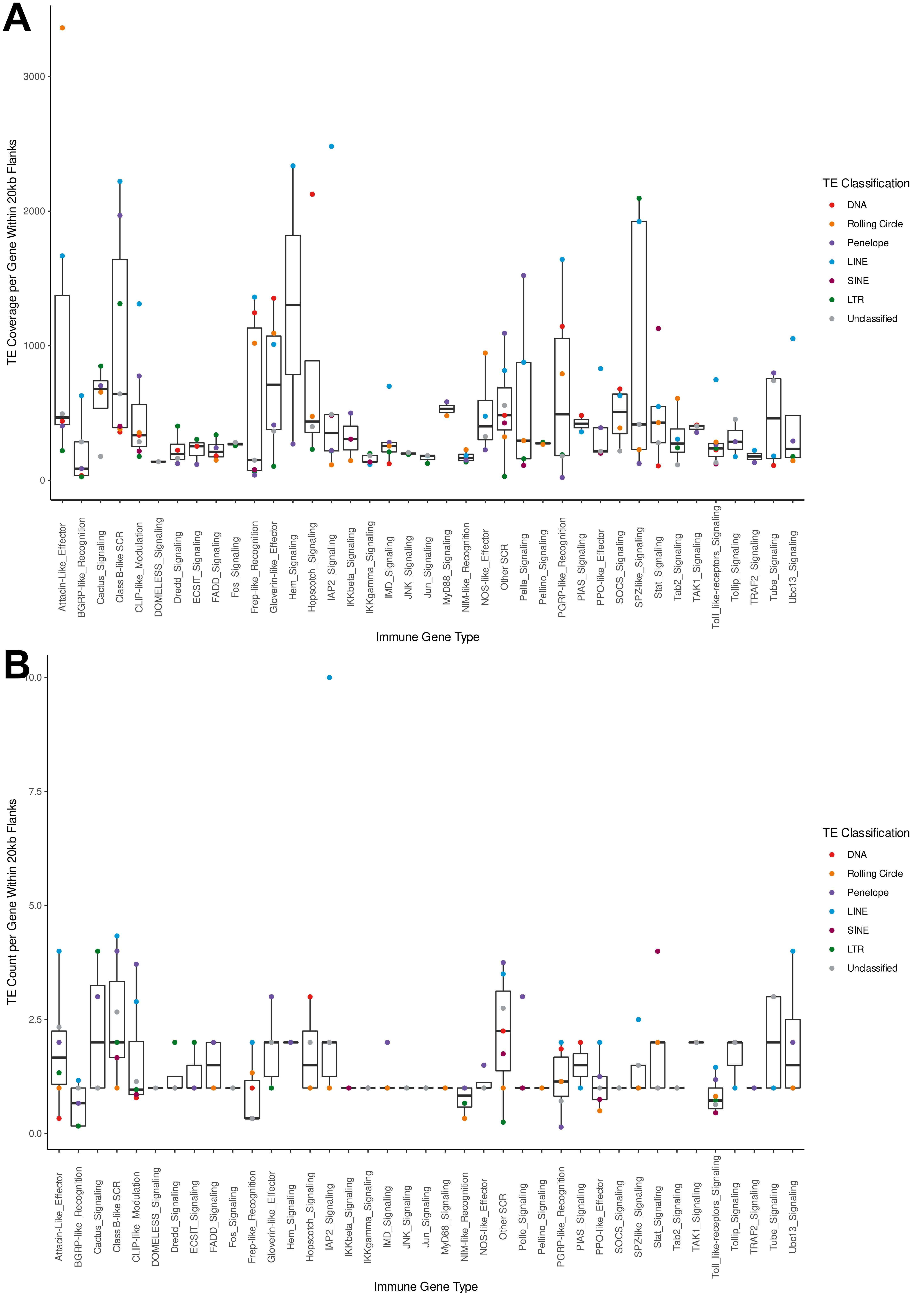
A
B
TE Coverage per Gene Within 20kb Flanks
TE Count per Gene Within 20kb Flanks
Immune Gene Type
TE Classification
DNA
Rolling Circle
Penelope
LINE
SINE
LTR
Unclassified
Attacin-Like_Effector
BGRP-like_Recognition
Cactus_Signaling
Class B-like SCR
CLIP-like_Modulation
DOMELESS_Signaling
Dredd_Signaling
ECSIT_Signaling
FADD_Signaling
Fos_Signaling
Frep-like_Recognition
Gloverin-like_Effector
Hem_Signaling
Hopscotch_Signaling
IAP2_Signaling
IKKbeta_Signaling
IKKgamma_Signaling
IMD_Signaling
JNK_Signaling
Jun_Signaling
MyD88_Signaling
NIM-like_Recognition
NOS-like_Effector
Other SCR
Pelle_Signaling
Pellino_Signaling
PGRP-like_Recognition
PIAS_Signaling
PPO-like_Effector
SOCS_Signaling
SPZ-like_Signaling
Stat_Signaling
Tab2_Signaling
TAK1_Signaling
Toll_like-receptors_Signaling
Tollip_Signaling
TRAF2_Signaling
Tube_Signaling
Ubc13_Signaling
