# Supplementary material for: Migrators within migrators: exploring transposable element dynamics in the monarch butterfly, Danaus plexippus
Source: Mob DNA. 2022 Feb 16;13:5. doi: 10.1186/s13100-022-00263-5 (PMC8848866; doi:10.1186/s13100-022-00263-5)

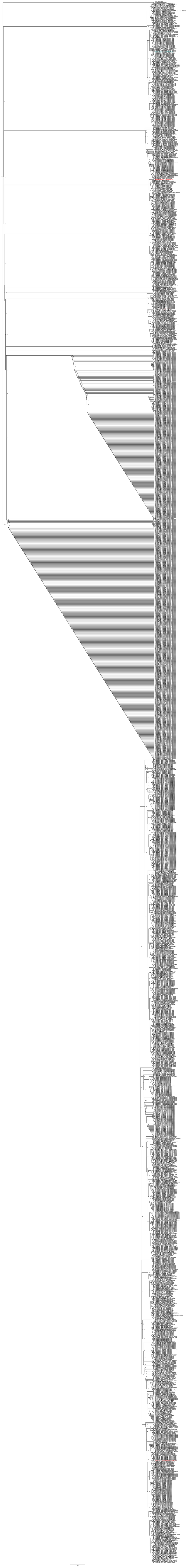

Supplement: Supplementary file 9 — Additional file 9: Fig. S7. Phylogenetic tree of Tc1-Mariner DDE transposases, with novel monarch families added (highlighted in red). Branch support from 1000 ultra-fast bootstrap repetitions. Novel Tc1-Mariner families highlighted in teal are the young TEs found with extremely high copy number. [file 13100_2022_263_MOESM9_ESM.pdf]
